# The combined effects of ethanolic extract of *Artemisia aucheri* and *Artemisia oliveriana* on biofilm genes expression of methicillin resistant *Staphylococcus aureus*

**Published:** 2018-12

**Authors:** Ghazaleh Shojaei Baghini, Abbas Akhavan Sepahi, Robab Rafiei Tabatabaei, Kambiz Tahvildari

**Affiliations:** 1 Department of Microbiology, Faculty of Basic Sciences, North Tehran Branch, Islamic Azad University, Tehran, Iran; 2 Department of Chemistry, North Tehran Branch, Islamic Azad University, Tehran, Iran

**Keywords:** *Artemisia aucheri*, *Artemisia oliveriana*, Biofilm, Gene expression, *Staphylococcus aureus*

## Abstract

**Background and Objectives:**

One of the most important antibiotic-resistant bacteria is methicillin-resistant *Staphylococcus aureus* (MRSA) biofilm that has caused significant problems in treating the patients. Therefore, the aim of this study was to evaluate the levels of expression of genes involved in biofilm formation in MRSA (ATCC 33591) while being treated by a combination of *Artemisia aucheri* and *Artemisia oliveriana*.

**Materials and Methods:**

The minimum inhibitory concentration (MIC) of ethanolic extract of *A. aucheri* and *A. oliveriana* and also the minimum inhibitory concentration of combination of both extracts were 512, 1024 and 256 μg/ml, respectively; then at concentrations lower than the MIC, expression levels of the desired genes were determined by Real Time PCR.

**Results:**

Based on results, using a combination of two ethanolic extracts had a significant effect on expression of genes involved in biofilm formation in MRSA. The expression level of *icaA* at 4, 8, 16 h after being treated by herbal extracts of *A. aucheri* and *A. oliveriana* was 0.293, 0.121, 0.044, respectively. The expression level of *icaD* was 0.285, 0.097, 0.088, respectively, while that of *ebps* was 0.087, 0.042, 0.009 at 4, 8 and 16 h, respectively.

**Conclusion:**

This study provided evidence that ethanol extract of *A. oliveriava* and *A. aucheri* can inhibit the biofilm formation of *S. aureus*. As a traditional Iranian medicine, *A. oliveriava* and *A. aucheri* extracts have a potential antibiofilm formation against MRSA strains.

## INTRODUCTION

*Staphylococcus aureus* is one of the most important microorganisms involved in general and hospital-acquired infections. This species is often able to develop multiple antibiotic resistancy and due to this ability, they can tolerate the process of treatment. Infection caused by methicillin-resistant *S. aureus*, is accompanied with increased probability of treatment failure and also economic loss (1, 2). The rate of mortality and contagiousness caused by general infections methicillin-resistant *S. aureus*, is significant like hospital-acquired infections caused by this strain (3).

The most important factor in the pathogenesis of *S. aureus* which is related to medical devices and equipment is its ability to form multiple-layers and sticky biofilms. Biofilms are organized communities of bacteria that are able to stick to the surfaces and consist of complex compounds like exopolysaccharides (EPS), nucleic acids and proteins (4). Biofilm development can be a reason behind the false negative culture result of some clinical specimen while bacterial infection exists in the specimen (5). Over-production of these compounds in a short period of time causes an increase in bacterial mass and finally prevents them from being killed by neutrophils (6). Additionally, biofilm formation is a key mechanism in the inhibiting effectiveness of antibiotics used for the treatment of staphylococcal infections (7).

Studies have shown that natural antimicrobial compounds such as herbal extracts affect bacterial antibiotic-resistant biofilms like biofilm formed by MRSA. The hydrophobic nature of these extracts facilitate the permeation into bacterial cell membrane lipids and consequently increases its permeability and this function leads to impairment in all cellular activity related to cell membrane, departure of ions from the cell and finally cell death (8).

Consumption of antibiotics often causes bacterial resistance against these drugs and led to some side effects in the patients. Due to these negative effects of antibiotics, using medicinal plants can be useful in improving the methods of treatment (9). *Artemisia* is a large and diverse genus of plant that can be found in northern temperate regions. Thirty four species of *Artemisia* have been found in Iran and several of its species have medicinal importance and are used in traditional herbal medicine for treatment of different kinds of diseases. The common important antimicrobial compounds isolated from different species of this plant are 1, 8 cineol and camphor (10). Terpene compounds also have antimicrobial properties, as the antibacterial activity of *A. fragrance* essence is reported (11–13).

Results of antibacterial assessment of essence of this plant showed that it has antibacterial activities against Gram positive bacteria such as *Streptococcus agalactiae, Enterococcus faecalis* and *S. aureus*, but it doesn't have any antibacterial effect on Gram negative bacteria such as *E. coli* and *Klebsiella pneumoniae*.

According to the previous studies conducted in the field of antibacterial properties of *Artemisia* species, the aim of this study was to determine the antimicrobial effects of *A. aucheri* and *A. oliveriana* on the expression of genes involved in biofilm formation in MRSA.

## MATERIALS AND METHODS

### Bacterial strain and culture conditions.

This work was conducted with the aim of evaluating combinational antibacterial effects of *A. aucheri* and *A. oliveriana* on expression level of genes involved in biofilm formation. In this study, standard strain of ethicillin-resistant *S. aureus* (ATCC 33591) was received from Pasteur Institute of Iran. As the first step, the strain was cultured on MHA (Muller Hinton Agar) and in MHB (Muller Hinton Broth) and was incubated at 37°C for 24 h and the isolate was used for subsequent evaluations.

### Preparations of plant extracts.

*A. aucheri* and *A. oliveriana* were prepared from Iranian Biological Resource Center. Ethanolic extracts of both herbs were obtained by maceration method. Extraction was done by soaking 20 g of the herb in 300 ml solvent (ethanol 80%) for 6 days and after that, filtration was done using Whatman filter paper and then the filtrate was placed in the room temperature till complete evaporation (14). To prepare the first stock of the herb extract, 0.5 g of herb extract was dissolved in 10 ml of normal saline. It should be mentioned that obtained extracts were placed in a container covered with aluminum layer to prevent oxidation.

### Determination of minimum inhibitory concentration (MIC).

To determine the antibacterial properties of ethanolic extracts of *A. aucheri* and *A. oliveriana*, microdilution method was used. In this method, minimum inhibitory concentration (MIC) was determined based on the method described by Nuriyastushi et al. with minor modifications (15). To prepare bacterial suspensions, one single colony from 24 h grown culture of MRSA on MHA was picked and dissolved in MHB. Then optical density was adjusted at 600 nm wavelength (LabTech - Model UV 9100 Series - UV/Vis Spectrophotometer) in such a way that equivalent turbidity was equal to 0.5 McFarland standard concentrations. Initially, serial dilutions were prepared for the herbal extracts from 4.96 to 5 mg/ml for both extracts. At first in each well of the microtiter plate, 95 microliter culture media was added and then 45 microliter herbal extract was added to the well that had 95 microliter culture media. Subsequently, 75 μl from this well was added to the second well and this action was repeated to the last well of the microtiter plate and 70 μl from the last was removed. Thereafter, 5 μl of microbial suspension (concentration equivalent to 0.5 McFarland standard = 1.5 × 10^8^ /ml) was added to the well except the one that didn't have any bacterial culture in it and was defined as our negative control (culture media + herbal extract) while the last well was our positive control (culture media + MRSA culture). Then, it was incubated at 37°C for 24 h. After this period of incubation, turbidity was measured in comparison with the positive control well showed bacterial growth. The minimum inhibitory concentration at which no growth was observed, was defined MIC. All experiments were repeated three times. MRSA culture was exposed to one concentration less than MIC of the herbal extracts at 4, 6, 8 h for treatment and RNA extraction.

### Quantification of cell viability in biofilm layer.

For evaluation of cell viability which treated with extracts and bacteriocin, the metabolic assay was used according to reference protocol. Briefly, the level of metabolic activity was measured with incorporating phenol red as pH indicator which change the color of MRSA culture media, when the strain produced an amount of metabolites. For quantification, we used the statistical formula which mentioned in Welch method (16).

### RNA isolation.

The RNA was extracted from MRSA culture by kit RNAPlus (Cinagen, Iran), then concentration and OD (optical density) of the extracted RNAs was measured by Nanodrop (Thermofisher, USA).

### cDNA preparation.

In order to prepare cDNA from the extracted RNA, a special kit named Revert Aid First Strand (cDNA synthesis kit, TAKARA, Japan) was used.

### Quantitative real-time PCR.

To evaluate the level of expression of *icaA, icaD, ebps* genes, qPCR was used. To define the performance of used primers, cDNAs of genes involved in biofilm formation were diluted and for each dilution, qPCR was done, then by drawing curve and determining the slope of the line, performance of each primer was calculated (17).

### Analysis of genes expression using Real-time PCR.

In this study, *icaA, icaD* and *ebps* genes were selected as target genes, *gyrB* as the housekeeping gene, and *MecA* as confirmatory gene for methicillin-resistant strains. Relevant primers were designed according to the Table 1.

**Table 1.** Sequences of oligonucleotide primers used for qPCR.

**Genes**	**Nucleotide sequence of primers (5′- 3′)**	**Annealing temperature**	**Amplicon size (bp)**
*icaA*	F : GAGGTAAAGCCAACGCACTC	60.5	151
	R; CCTGTAACCGCACCAAGTTT	58.4	
*icaD*	F; ACCCAACGCTAAAATCATCG	56.4	211
	R; GCGAAAATGCCCATAGTTTC	56.4	
*ebps*	F; GGTGCAGCTGGTGCAATGGGTGT	68.3	191
	R; GCTGCGCCTAGCCAAACCT	67.3	
*mecA*	F; GTC AAG ATA TAC CAA GTG ATT	53.5	147
	R; ATG CGC TAT AGA TTG AAA GGA T	56.6	
*gyrB*	F; AGT AAC GGA TAA CGG ACG TGG TA	62.9	147
	R; CCA ACA CCA TGT AAA CCA CCA GAT	63.5	

Total RNA of treated and untreated microbial strains were extracted using an RNA extraction kit (RNX-Plus kit, CinaGen Company) according to manufacturer's instructions. The quantity and quality of extracted RNAs were determined using Nanodrop BioTek Epoch (USA) and agarose gel electrophoresis.

Afterwards, cDNA synthesis was performed using a cDNA synthesis kit (Takara, Japan) based on manufacturer's instructions. Finally, the gene expression level was evaluated using Real-time PCR (ABI model) according to the following program: 95°C (15 min), 95°C (30 sec), and 60°C for 1 min during 40 cycles.

### Statistical analysis.

Finally, the authenticity of amplified products were confirmed by melting curves for each product and melting curve was drawn three times. Data analysis was performed by Relative expression software (REST2009) and SPSS (Version 21).

## RESULTS

The level of susceptibility of MRSA ATCC33591 to combinational effects of ethanolic extracts of *A. aucheri* and *A. oliveriana* was determined quantifiably by MIC method. The minimum inhibitory concentration of ethanolic extract of *A. aucheri* and *A. oliveriana* and also the minimum inhibitory concentration of combination of both extracts were 512, 1024 and 256 μg/ml, respectively. The expression level of genes involved in biofilm formation (*icaA, icaD* and *epbs*) had statistically significant (p< 0.001) in comparison with housekeeping gene (*gyrB*) at 4, 8, 16 h after being treated by herbal extracts of *A. aucheri* and *A. oliveriana.*

The expression level of related genes are shown in Table 2. In fact, the levels of expression of studied genes decreased after mentioned hours. The relative expression report of following the treatment with *A. aucheri* and *A. oliveriana* is shown in Table 2.

**Table 2.** Relative expression report of using *Artemisia aucheri* and *Artemisia oliveriana*

**Hour**	**Gene**	**Type**	**Reaction Efficiency**	**Expression**	**Std. Error**	**95% C.I.**	**P (H1)**	**Result**
	*gyrB*	REF	1.0	1.000				
4	*icaA*	TRG	1.0	0.293	0.203 - 0.443	0.165 - 0.616	0.000	DOWN
8	*icaA*	TRG	1.0	0.121	0.095 - 0.154	0.063 - 0.183	0.000	DOWN
16	*icaA*	TRG	1.0	0.044	0.031 - 0.059	0.027 - 0.076	0.000	DOWN
4	*icaD*	TRG	1.0	0.285	0.229 - 0.354	0.177 - 0.435	0.000	DOWN
8	*icaD*	TRG	1.0	0.097	0.072 - 0.134	0.063 - 0.181	0.000	DOWN
16	*icaD*	TRG	1.0	0.088	0.047 - 0.154	0.036 - 0.204	0.000	DOWN
4	*ebps*	TRG	1.0	0.087	0.051 - 0.154	0.041 - 0.287	0.000	DOWN
8	*ebps*	TRG	1.0	0.042	0.024 - 0.077	0.017 - 0.095	0.000	DOWN
16	*ebps*	TRG	1.0	0.009	0.007 - 0.010	0.006 - 0.013	0.000	DOWN

P (H1): Probability of alternate hypothesis that difference between sample and control groups is due only to chance. It showed p value for each genes.

TRG : Target. REF : Reference.

The biofilms of MRSA retained 2–100% cell viability following treatment with ethanolic extract of *A. aucheri, A. oliveriana* and mix of them at 4, 8, 16 h as shown in Fig. 3.

**Fig. 1.**
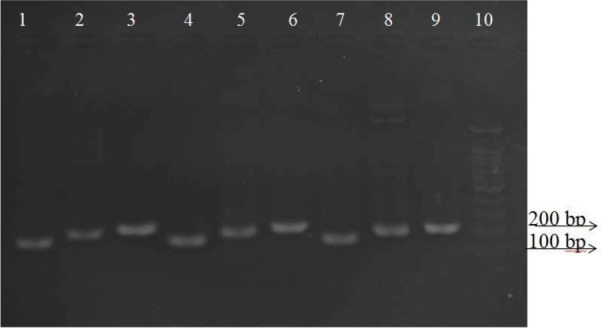
Confirming the existence of desired genes in the strain. Columns 1, 4, 7: amplified PCR products of *icaA* gene (151 bp) Columns 2, 5, 8: amplified PCR products of *ebps* gene (191bp) Columns 3, 6, 9: amplified PCR products of *icaD* gene (211 bp) Column 10: marker

The results of gel electrophoresis of PCR products of *icaA, icaD* and *epbs* genes on agarose gel are shown in Fig. 1. It cleared all primers work perfect and specific.

**Fig. 2.**
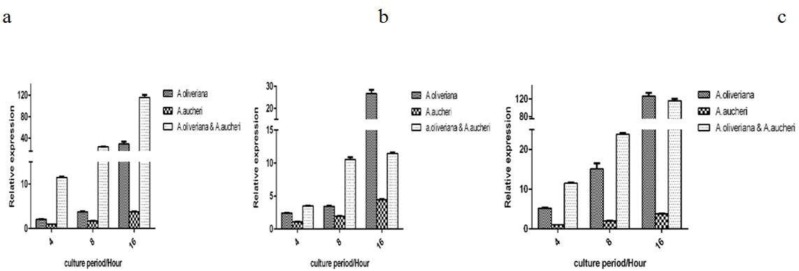
Comparison of average decrease in gene expression in three medicinal groups: a) *icaA* group, b) *icaD* group, C) *ebps* group

Average (SEM: Standard Error of Mean) level of expression of genes required for biofilm formation (*icaA, icaD* and *epbs*) at 4, 8 and 16 h after being treated by ethanolic extracts of *A. aucheri* and *A. oliveriana* and combination of both extracts is shown in Fig. 2.

**Fig. 3.**
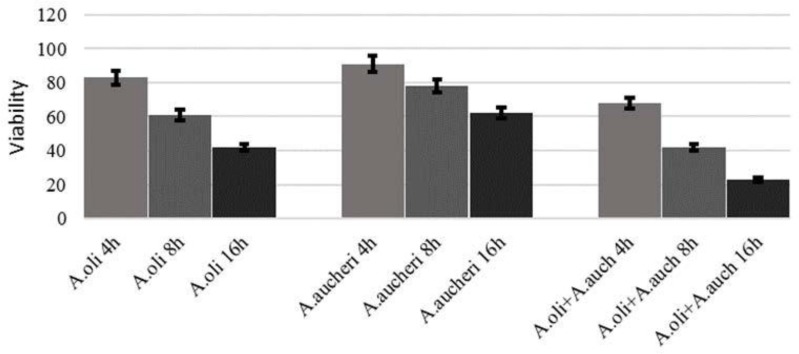
Cell viability (%) in biofilms of MRSA following treatment with antibacterial plant extract

## DISCUSSION

According to increasing spread of antibiotic resistance among pathogenic bacteria, it seems that the most promising solution for treating these kinds of infections is using natural compounds like medicinal herbs. By now, many studies have been conducted on this issue and on the effects of medicinal herbs on bacteria including *H. pylori, S. aureus, E. coli, Pseudomonas aeroginosa* and many other bacteria and this study has proven that medicinal herbs such as cinnamon, green tea, carnation, khuzestani chives and many others by making changes in bacterial cell wall and inhibiting their growth (18, 19).

Overuse of antibiotics causes financial loss and waste of financial sources as it includes 20–50% of the entire hospital drug expenditure (20). According to the importance of herbal compounds in treatment of bacterial infections and also increased attention by researchers towards them, several recent studies in different fields of controlling biofilm by these compounds have been mentioned.

In this study, MRSA strain that had *icaA, icaD* and *epbs* genes was studied and the results obtained confirmed that applying extracts of *A. oliveriana* + *A. aucheri* together as a combination had the potential of inhibiting genes involved in biofilm formation in methicillin-resistant *S. aureus* (ATCC 33591).

In Yang Chio's study in 2015, the effects of *Artemisia princeps* ethanolic extract on MRSA was evaluated and results of this study were expressed as inhibition of genes involved in pathogenesis, biofilm formation and acid production (12). The respected genes were analyzed in Chio's study were *sea, agrA* and *sarA* and have different with our target genes. Moreover, the effective dose of Artemisia princeps extract in his survey was 2 mg/ml.

In 2016, Fan Zhang et al. studies indicated the antimicrobial effects of peppermint essence and Nicin on growth of *E. coli* and *Listeria monocytogenes* and combination effects of peppermint and bacteriocin (Nicin) on planktonic cells of both bacteria were confirmed (19). In current study, the combination effects of extracts and bacteriocin was more on biofilm down regulating genes expression instead of individual respected mixture.

In Saddiq et al. study in 2018, the effects of the antibacterial impact of Aloe vera leaf aqueous extract against six strains of methicillin resistant *S. aureus*. The results revealed that the plant extract at concentrations of 15–20 mg/ml, markedly reduced the dry weights of most *S. aureus* strains after 24 and/or 48 h exposure periods (21). In our study, down regulation of target genes expression were more and showed these extract have more potent effect of biofilm formation among MRSA. In our study, down regulation of target genes expression were more and showed these extract have more potent effect of biofilm formation among MRSA.

In total, based on the results obtained in this study, it can be concluded that extracts of medicinal herbs *A. oliveriana* and *A. aucheri* have effective compounds for inhibiting expression of genes involved in biofilm formation in MRSA.

## CONCLUSION

One of the most important strategies of microbial pathogenesis is biofilm formation which lots of gene are involved. On the other hand, these genes are suitable targets for new component could affect their expression. So, the scientists are interested to assess various natural and synthetic components effects on biofilm gene expression. Our survey showed respected herbal extracts and nicin have a potent effect on genes which involved biofilm formation in MRSA strain and could be notice for more evaluation.
